# Metabolome dynamics during wheat domestication

**DOI:** 10.1038/s41598-022-11952-9

**Published:** 2022-05-20

**Authors:** Yuval Ben-Abu, Mark Itsko

**Affiliations:** 1 Department of Physics and Project Unit, Sapir Academic College, 79165 Sderot, Hof Ashkelon, Israel; 2 Clarendon Laboratory, Department of Physics, University of Oxford, Oxford, UK; 3 Present Address: WDS Inc., Contractor to Centers for Disease Control and Prevention, 1600 Clifton Road, Atlanta, GA 30033 USA

**Keywords:** Experimental evolution, Ecology, Evolution, Plant sciences

## Abstract

One of the most important crops worldwide is wheat. Wheat domestication took place about 10,000 years ago. Not only that its wild progenitors have been discovered and phenotypically characterized, but their genomes were also sequenced and compared to modern wheat. While comparative genomics is essential to track genes that contribute to improvement in crop yield, comparative analyses of functional biological end-products, such as metabolites, are still lacking. With the advent of rigorous mass-spectrometry technologies, it is now possible to address that problem on a big-data scale. In attempt to reveal classes of metabolites, which are associated with wheat domestication, we analyzed the metabolomes of wheat kernel samples from various wheat lines. These wheat lines represented subspecies of tetraploid wheat along primary and secondary domestications, including wild emmer, domesticated emmer, landraces durum, and modern durum. We detected that the groups of plant metabolites such as plant-defense metabolites, antioxidants and plant hormones underwent significant changes during wheat domestication. Our data suggest that these metabolites may have contributed to the improvement in the agricultural fitness of wheat. Closer evaluation of specific metabolic pathways may result in the future in genetically-engineered high-yield crops.

## Introduction

To feed the rapidly growing global population, the scientific community invests intensive efforts in improving the yield and nutritional values of crops, among which is the modern wheat^1,2^ Modern agriculture technologies and genetic modification techniques are being constantly improved^3^. Agricultural technology advancement aside, it is crucial to identify which crop traits should be improved, and how to specifically and effectively target them. One of the ways to face that challenge is to investigate crop evolution and domestication. This 10,000-years-long process has eventually resulted in wheat strains far more adapted for human needs, including larger grain size, increase grain number, grain retention and thresh ability required for efficient harvest, high yield, altered photoperiod sensitivity, and a higher glycemic load^1,2^. These phenotypic traits are a collective endpoint result of a highly complex regulatory genetic network. In between, numerous proteins and metabolites carry out their biological function, creating a vast and diverse biochemical network. Thus, the analytical measurement of these bioactive molecules and their intermediates enables us to functionally link between the plant genotype and the plant phenotype. In the recent decade, analytic “Omics” approaches have become more popular to compare between two or more physiological conditions, between different tissues or between species, consequently producing extensive datasets. Not only that the genomes of modern bread wheat (*Triticum aestivum*) and durum wheat strains (*Triticum turgidum *ssp.* durum*) have been sequenced^4–6^, but also the genomes of their progenitors, including domesticated emmer (*Triticum turgidum *ssp.* dicoccum*) and wild emmer (*Triticum turgidum* ssp.* dicoccoides*), have been sequenced and compared^7–9^. Many quantitative trait loci and genetic polymorphism markers, which distinguish between modern and wild wheat strains, have been identified and characterized^1,4,6,10–12^. While comparative studies, which utilize genomics, transcriptomics, or proteomics, are quite common^13^, mass-spectrometry (MS)-coupled metabolomics is not commonly used in crop sciences^14^. Nevertheless, metabolomics expand our knowledge about how evolutionary genetics during wheat domestication is translated into changes in fluxes of metabolites found in plants^14,15^. Several recent studies have employed this methodology, for example, to compare between wheat lines^16,17^, or to evaluate environmental stress-response in wheat^18–20^. Employment of metabolomics to investigate wheat domestication is still uncommon. So far, three metabolomics-based studies have compared between the tetraploid wild emmer, emmer and durum wheat strains: one study analyzed the root exudates^21^, the second study analyzed the kernels^22,23^, the third study analyzed changes in specific group of metabolites playing important role in protection of the seeds from a variety of biotic stresses^24^. In this study we compared ancestor wheat strain wild emmer (WEW, *Triticum turgidum* ssp.* dicoccoides*) with its early domesticated version (DEW, *Triticum turgidum *ssp.* dicoccum*) and later selected durum wheat (*Triticum turgidum *ssp.* durum*), classified into ancient landraces of durum (LD) which are traditional crops, and modern durum (MD) representing high-yielding industrial crops. All those strains are tetraploides. We did not include in this study hexaploid modern bread wheat lines (*Triticum aestivum*) to avoid unnecessary complexity in comparison with the tetraploid strains. We employed MS-coupled metabolomics with respect to wheat domestication, taking it one step further. In comparison with the previous studies we added more variables, by separating the kernel embryo and endosperm, by creating four categories of wheat strains instead of three, and by increasing the pool of identified metabolites.

## Materials and methods

### Statement on experimental research on plants

The producers granted permission for plant and seed measurements, which were done in situ. All methods and assays performed in this field study comply with national legislation and guidelines of the Sapir Academic College and Weizmann institute of science, Israel.

Wild emmer seeds wheat (*T. turgidum* ssp. *dicoccoides*) and the others type accessions used in this study were provided to us with permission from the collection by Israeli stock centers, as following: Wild Cereals gene bank (WCGB)—Prof Eibi Nevo, Institute of Evolution, University of Haifa and Prof Moshe Feldman, the grain wheat bank of Weizmann institute of science, Israel.

### Plant material

Nineteen wheat lines that correspond to various stages of wheat domestication at the tetraploid level were grown and analyzed (4–5 grains in each line). They consist of wild emmer (WE, *Triticum turgidum* ssp.* dicoccoides*), domesticated primitive emmer (PE, *Triticum turgidum *ssp.* dicoccum*), domesticated landraces varieties of durum (LD), and modern durum (MD) (*Triticum turgidum *ssp.* durum*). The PE and LD lines were collected from traditional farmers, whereas the MD lines represent high-yielding macaroni wheat. The annotations of the wheat lines and their geographical origin are shown in Table 1. The plants were grown under the same conditions, in a net-house with three replicates per line; each replicate being grown in a separate block. All plants were grown in 3-L pots during the winter^25^.

**Table 1:** Tetraploid wheat lines used in this study (origin).

Wild Emmer (WE) *Triticum turgidum* ssp. dicoccoides	Primitive Emmer (PE) *Triticum turgidum* ssp. dicoccum	Landraces Durum (LD) *Triticum turgidum* ssp. durum	Modern Durum (MD) *Triticum turgidum* ssp. durum
TTD20 (Israel)	Sub-group A: TTC1 (Italy)	TTR265 (Israel)	TTR16 (USA)
TTD32 (Turkey)	Sub-group A: TTC2 (India)	TTR2 (Israel)	TTR19 (Italy)
TTD43 (USA)	Sub-group A: TTC8 (India)	TTR333 (Turkey)	TTR1 (Portugal)
TTD49 (Israel)	Sub-group B: TTC4 (Nigro-ajar)	TTR5 (Morroco and Tunisia)	TTR25 (Israel)
TTD68 (Israel)	Sub-group B: TTC6 (origin unknown)	TTR6 (Israel)	

### Sample preparation

Whole grain samples, separated into endosperm and embryo, were used for the study. The metabolite extraction was performed as described previously^24^. In brief, the embryo and endosperm tissues samples (500 mg) of different lines were ground to a fine powder in liquid nitrogen, and 1.5 mL of 75% methanol with 0.1% formic acid was added. After sonication at room temperature for 15 min, the samples were centrifuged (10,000*g*) and filtered. The samples were stored at − 20 °C prior to analysis. The metabolites in the extractable fraction were further purified by column chromatography (Amberlite XAD 8 HP) and eluted with ethanol. Both fractions were freeze-dried and stored at − 80 °C.

### LC–MS metabolite analysis

Metabolite analysis was carried out using as described by Hanhineva et al. (2008, 2011) and by Ben Abu et al. (2021)^24,25^. This procedure used a UPLC-PDA-qTOF-MS system: a UPLC Waters Acquity instrument connected in-line to an Acquity PDA (photodiode array) detector and a Synapt HDMS detector (tandem quadrupole/time-of-flight mass spectrometer). This analysis was carried out using ultra-performance liquid chromatography coupled with a photodiode detector-quadrupole and tandem time-of-flight mass spectrometry (UPLC-PDA-qTOF-MS-Waters Premier qTOF, Milford, MA, USA). The system consisted of a Acquity UPLC (Waters) connected in-line to an Acquity PDA detector (Waters) and a Synapt HDMS detector (Waters). The HDMS system was operated in the standard qTOF mode, without using the ion mobility capabilities. Metabolite separation was performed using a UPLC BEH C18 column (100 × 2.1 mm i.d., 1.7 μm; Waters). The mobile phase consisted of 0.1% formic acid in acetonitrile/water (5:95, v/v) (phase A) and 0.1% formic acid in acetonitrile (phase B). The linear gradient program was as follows: 100–72% A over 22 min, 72–60% A over 0.5 min, 60–0% A over 0.5 min, holding at 100% B for a 1.5 min, then returning to initial conditions (100% A) over 0.5 min, and conditioning at 100% A. The flow rate was 0.3 mL/min, and the column temperature was kept at 35 °C. The UV spectra were recorded at 210–550 nm using the Acquity PDA detector (Waters), or the UV trace was measured at 240 nm using the Acquity UV detector (Waters). Eluting compounds were detected using the qTOF equipped with an electrospray ionization (ESI) source. Acquisition was performed in ESI-positive and ESI-negative modes. The following settings were applied during the LC–MS runs: capillary voltage, 3.0 kV; cone voltage, 30 eV; collision energy, 3 eV and 20 eV; and collision gas, argon. For the LCMS/MS analysis, collision energies of 20 and 35 eV were used. The m/z range was 50–1500 Da. The MS was calibrated using sodium formate, and leucine enkephalin was used as the lock mass. A standard mixture containing 40 μg/mL of each of the following compounds was used to monitor the quality of the chromatogram, to ensure the consistency of retention times across runs, and to aid in metabolite identification: L-tryptophan, L-phenylalanine, p-coumaric acid, caffeic acid, sinapic acid, benzoic acid, quercetin dehydrate, kaempferol, rutin, and trans-resveratrol (all purchased from Sigma); naringenin, chlorogenic acid hemihydrate, trans-cinnamic acid, and isorhamnetin (Fluka); ferulic acid (Aldrich); and tomatine (Apin chemicals). Mass Lynx v4.1 (Waters) was used to control all instruments and to calculate accurate masses^7,26–30^.

### LC–MS data analysis

The chromatograms obtained from UPLC PDA-qTOF-MS analysis were processed by the Marker Lynx 4.1 software (Waters) for mass signal extraction and alignment. The identification of metabolites was performed as described previously on data obtained using the ESI-negative mode^24^. In short, the accurate mass and molecular formula predictions were screened for putative molecules from the Dictionary of Natural Products (Chapman and Hall/ CRC) and the SciFinder Scholar databases (SciFinder Scholar 2007) as described elsewhere^25^. The MS/MS fragmentation and UV-absorbability of the metabolites were compared with those of candidate molecules found in databases and verified with earlier literature on similar compounds^23^.

### Software and statistical analysis

Dataset and statistical analyses were done using Microsoft Excel 365, MATLAB 8.0 and Statistics Toolbox 8.1 software. p (FDR) < 0.05 is considered significant. We ascribed function to metabolites based on databases, and complementary internet search. Figures were generated using, MATLAB 8.0, GraphPad Prism 7.0 and Meta-Chart.

## Results

### Identification of metabolites associated with wheat domestication

**Figure 1:**
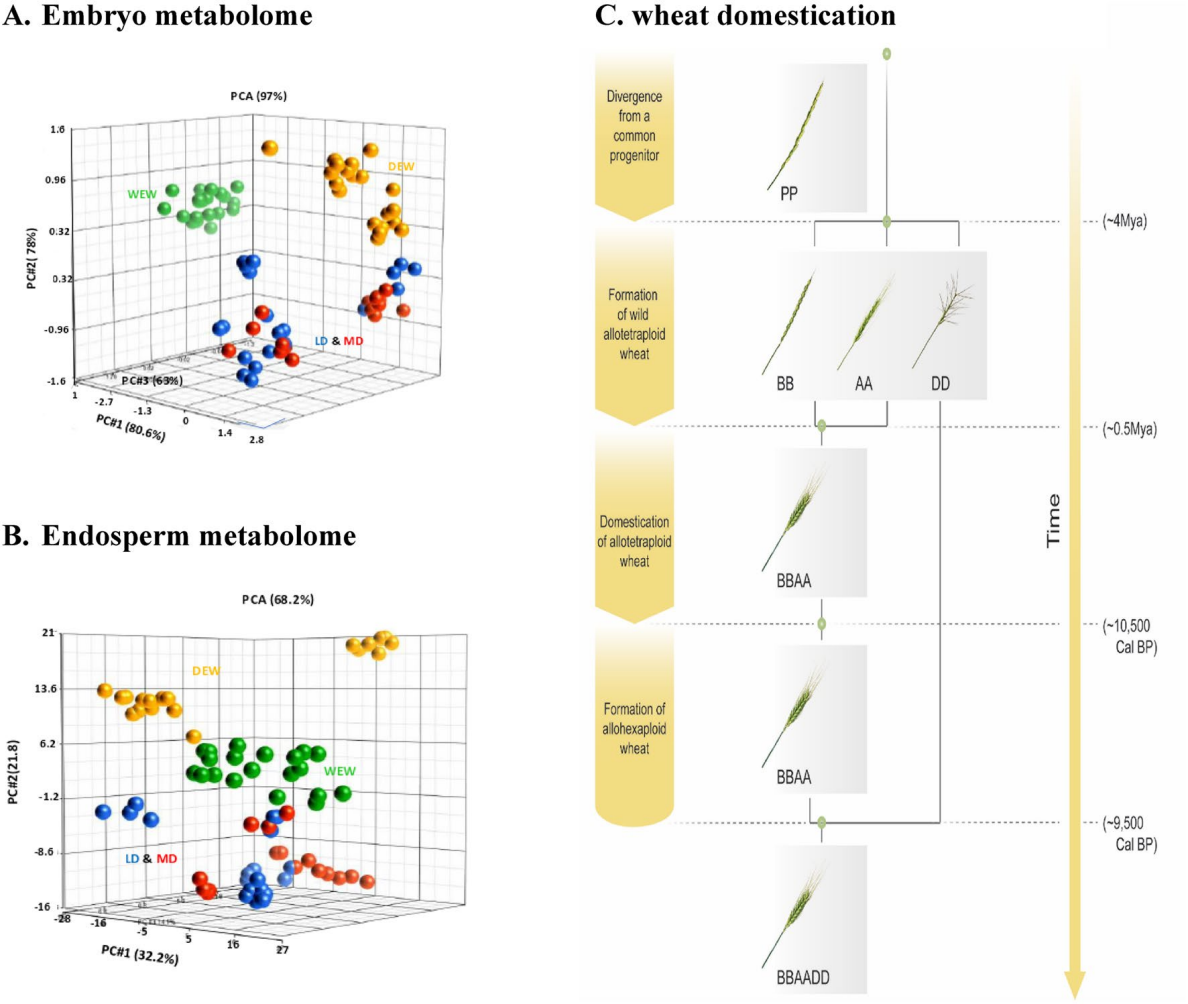
Three-dimensional models of principal component analyses (PCA). Each ball represents a quantile-normalized metabolome of one wheat line replica sample. (**A**) Embryo metabolomes—two perspectives. (**B**) Endosperm metabolomes—two perspectives. The dashed circles surround distinct groups of wheat metabolomes. WEW—wild emmer; WED (PE)—primitive emmer; LD—landraces durum; MD—modern durum. (**C**)**.** Evolutionary history of allotetraploid and allohexaploid wheat: Diploid wheats (2n = 2 ×  = 14), from the *Tritcum-Aegilops* group have diverged ~ 4Mya from a diploid progenitor whose genome is indicated here as PP. Intergeneric hybridization between the diploid *T. urartu* (genome AA) as male and the donor of BB genome as female, (an unknown species similar to Ae. speltoides), followed by chromosome doubling, gave rise (~ 0.5Mya) to the wild allotetraploid wheat, *Triticum turgidum*, ssp. dicoccoides (genome BBAA, 2n = 4 ×  = 28), the direct progenitor of durum and bread wheat. Domestication of allotetraploid wheat took place ~ 10,500 years ago and was followed by a second round of intergeneric hybridization chromosome doubling between domesticated allotetraploid wheat and the donor of the D genome, *Ae. Tauschii* (2n = 2 ×  = 14, genome DD), giving rise, ~ 9000 years ago, to bread wheat, an allohexaploid (2n = 6 ×  = 42, genome AABBDD).

**Figure 2:**
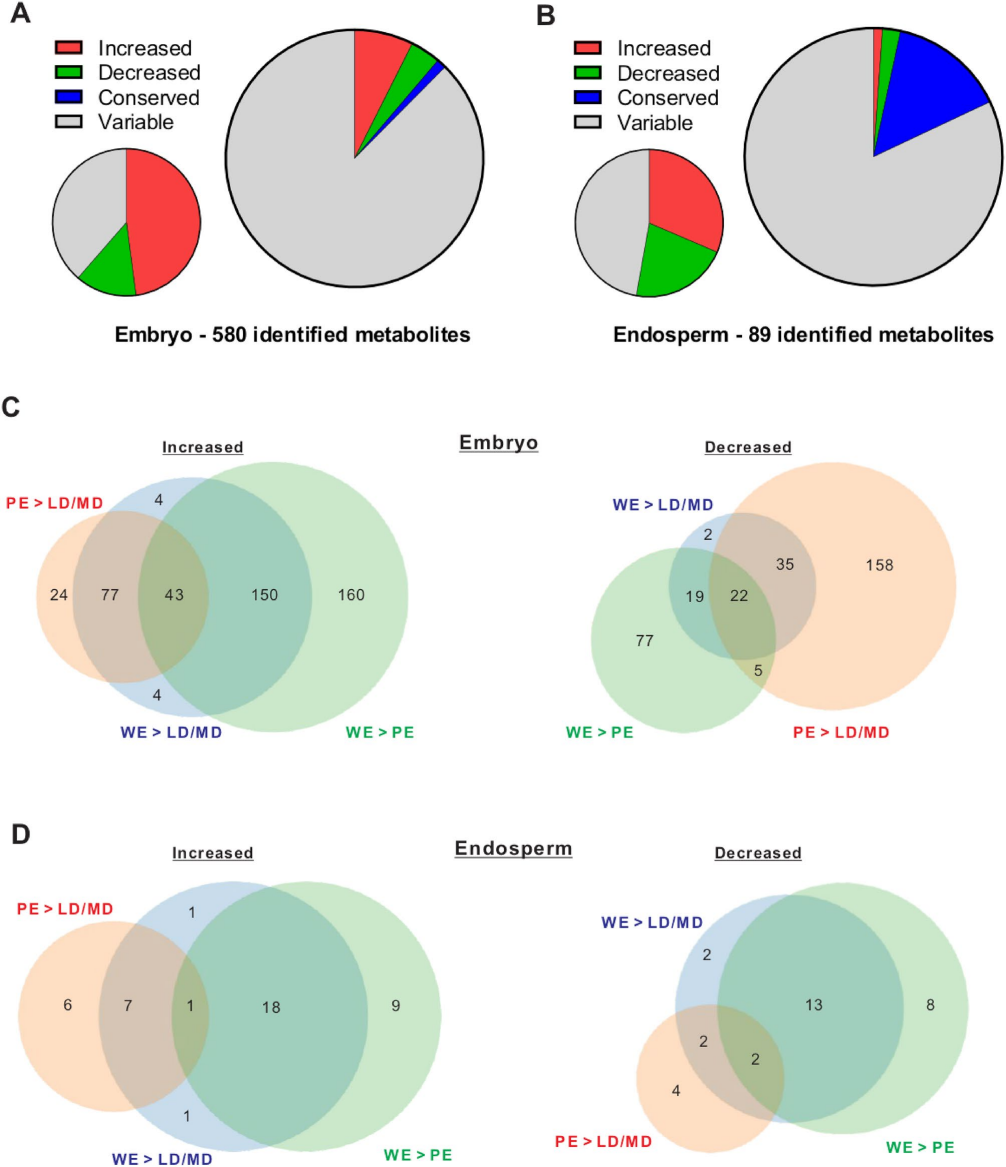
Comparative metabolomics. Identified metabolites that underwent significant changes in association with wheat domestication. Each metabolome is a result of group-averaged metabolite values from all the replicas and wheat lines that belong to that group. A statistical comparison is made between each pair of groups from a total of 4 groups: WEW—wild emmer; WED (PE)—primitive emmer; LD—landraces durum; MD—modern durum. (**A,C**) Comparison of embryo metabolomes. (**B,D**) Comparison of endosperm metabolomes. (**A-B**) Small pie charts—comparisons between the WE group and the LD/MD groups. Metabolites that underwent a significant increase or decrease in two comparisons (WE to LD and WE to MD). Variable metabolites either did not undergo statistically significant changes or did not increase or decrease in both comparisons. Large pie charts—comparisons between the WE, PE group and the LD/MD groups. Metabolites that underwent a significant increase or decrease in three comparisons (WE to PE, PE to LD and PE to MD) were considered to be steadily changed metabolites. Conserved metabolites did not undergo any statistical changes in any comparison. Variable metabolites did not undergo a steady increase or a steady decrease in all three comparisons. (**C-D**) Venn diagrams depict the numbers of statistically changed metabolites in the following comparisons: WE to PE, PE to both LD and MD, and WE to both LD and MD, and steadily changed metabolites. Increased metabolites on the left and decreased metabolites on the right.

We collected wild, primitive, and domesticated tetraploid wheat lines form various eco-geographical locations (Table 1). In order to associate metabolite profiling with wheat domestication, we classified these wheat lines into four categories (from wild lines to most domesticated lines): WEW, DEW, LD, MD. These wheat lines belong to three taxonomic subspecies of tetraploid *Triticum turgidum*, representing primary (WEW to DEW) and secondary (DEW to LD/MD) domestications^1^. To analyze specific trends, we identified metabolites present in wheat kernels using an annotated database^24^. In the embryo samples, we identified 580 metabolites, whereas in the endosperm samples, we identified 89 metabolites. We next attempted to characterize which metabolites undergo changes in association with wheat domestication. For that purpose, we undertook used PCA analysis as we perform elsewhere^25^ and appear in Fig. 1A,B. Moreover, we used two statistical approaches. First, we directly evaluated which identified metabolites significantly increase or decrease when the LD and MD groups are compared with the WEW group. In the embryo kernel, a total of 47.9% showed an increase, and a total of 13.5% showed a decrease (Fig. 2A—small pie chart). In the kernel endosperm, a total of 31.5% showed an increase, and a total of 21.4% showed a decrease (Fig. 2B—small pie chart). Secondly, we evaluated which metabolites steadily increase or decrease in both primary and secondary domestications (i.e., WEW to DEW and DEW to LD/MD respectively). Our analysis revealed that out of the identified metabolites in the kernel embryo 7.4% increased and 3.8% decreased in association with wheat domestication (Fig. 2A—large pie chart, Table S1). In the kernel endosperm, 1.1% increased, and 2.3% decreased in association with wheat domestication (Fig. 2B—large pie chart, Table S1). A very few metabolites remained conserved in the kernel embryo, while a relatively higher proportion of metabolites remained conserved in the kernel endosperm (Fig. 2A,B—large pie charts, Table S1). Breaking apart primary and secondary domestications revealed that many more metabolites underwent changes in the domestication steps considered here (Fig. 2C,D), therefore only a small portion of metabolites steadily changed over time (Fig. 2A,D). All the other metabolites were variable in their expression behavior. Together, these results suggest that the kernel embryo was subjected to a greater evolutionary pressure upon domestication. Considering the larger pool of metabolites detected in the embryo and the observation that metabolites in the endosperm did not undergo marked changes (fold-change-wise, not shown), we further focused on the embryo.

**Figure 3:**
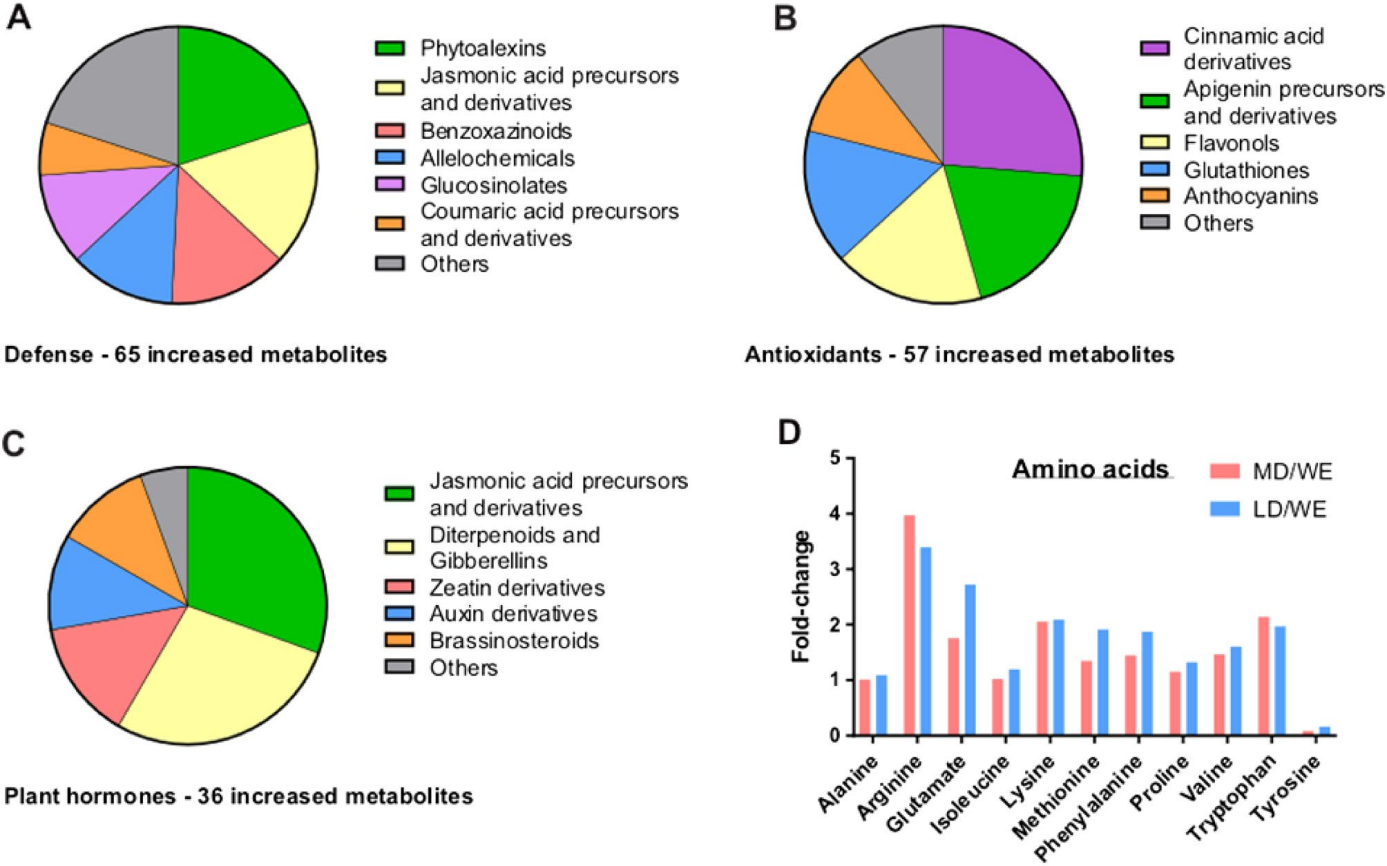
Categories of biological function. Embryo metabolomes. Identified metabolites that underwent a significant increase in association with wheat domestication (WE to LD and MD) were classified according to chemical structure and biological function. (**A**) Metabolites that are involved in plant defense mechanisms. (**B**) Metabolites that are known to be antioxidants. (**C**) Plant hormones and their intermediates. (**D**) Proteinogenic amino acids.

**Figure 4:**
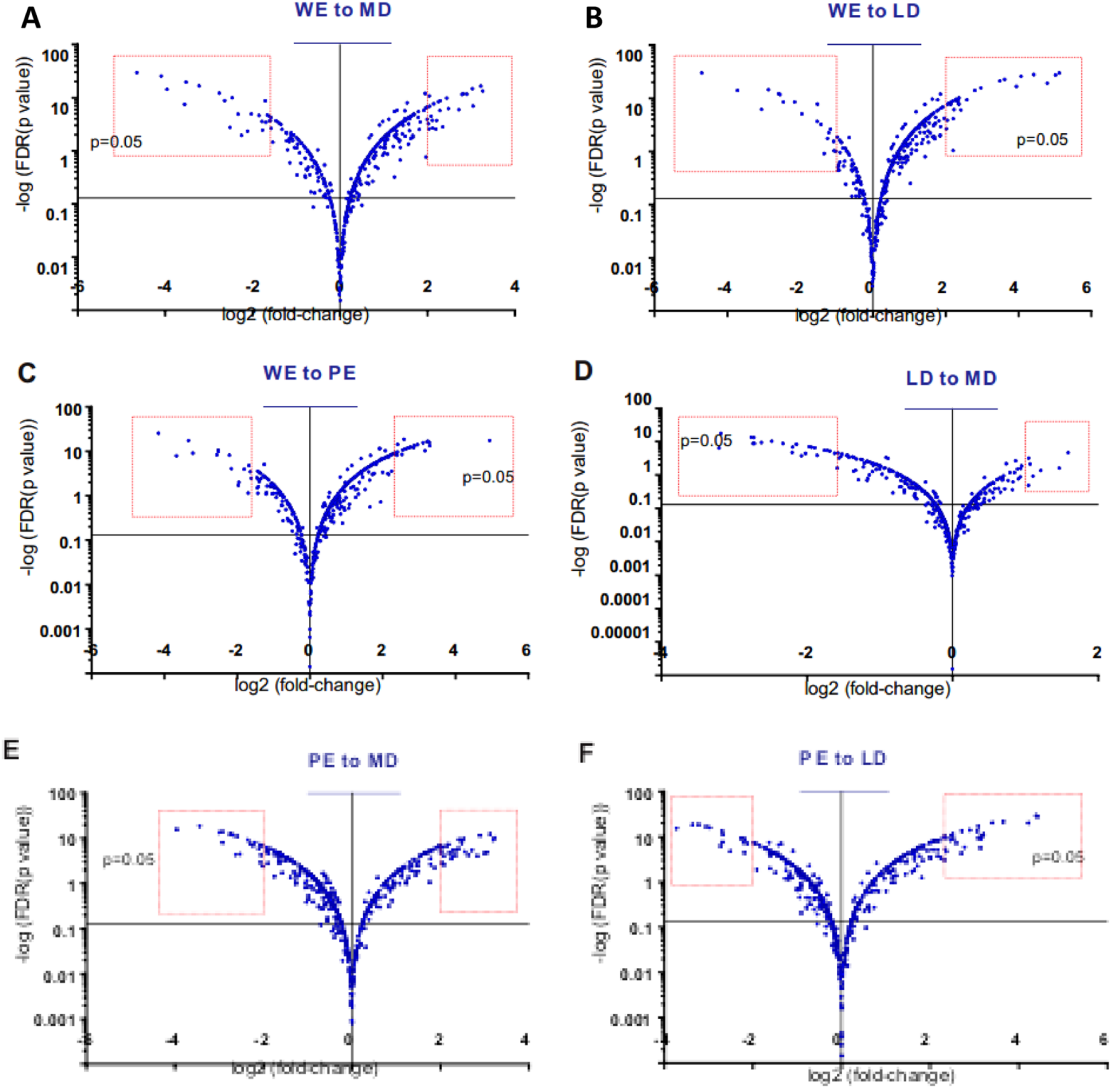
Volcano plots were generated based on comparative metabolomics between the average embryo metabolomes of each group (only identified metabolites, see also Fig. 3). WEW—wild emmer; DEW—primitive emmer; LD—landraces durum; MD—modern durum. All the metabolites that are above the horizontal line of *p* < 0.05 were considered significant. Red rectangles insets—the most significantly changed metabolites are shown in Tables S1 and S2.

Heat-maps show how certain metabolites in the kernel embryo, classified into 4 clusters, were co-regulated among wheat lines within the same group (Fig. S1), suggesting common co-regulated metabolic pathways. It is reasonable to assume that upon wheat domestication, the evolutionary pressure selected for certain biological functions and suppressed others. Thus, we classified the significantly changed metabolites into categories of biological function, using online databases (see “Materials and methods” section). Strikingly, the top-ranked category of increased metabolites consisted of compounds that are known to be involved directly or indirectly in plant defense mechanisms. This category comprised 23.4% of the increased metabolites (see also Fig. 2A—large pie chart) and 41.9% of the steadily increased metabolites (see also Fig. 2A—small pie chart). These compounds included phytoalexins, jasmonic acids, benzoxazinoids, glucosinolates, allelochemicals, coumaric acids, and others (Fig. 3A). The second and third top-ranked categories consisted of antioxidants and metabolites that are involved in growth and development (20.5% and 12.9% of increased metabolites, respectively). These compounds included cinnamic acid derivatives, apigenin derivatives, flavonols, glutathiones, anthocyanins, and several plant hormones (Fig. 3B,C). As for significantly decreased metabolites, we did not identify any outstanding category based on biological function. Metabolites that participate in amino acid metabolism was also evident among increased metabolites. The relative composition of amino acids in wheat kernels was therefore altered with domestication; we detected an increase in half of the amino acids and a decrease in one amino acid (Fig. 3D). In order to identify additional metabolites of interest, we generated six volcano plots, which took into account the fold-change and the statistical significance. Each volcano plot compares between a pair of group-averaged embryo metabolomes (Fig. 4A-F). The metabolites that underwent the most significant alterations are shown in Table S2 (also marked in Fig. 4A-F). Altogether, our findings demonstrated that upon wheat domestication, the wheat metabolome had been substantially altered. Follow-up research will determine how altered metabolic pathways and specific metabolites contributed to the fitness of modern wheat to agricultural needs.

## Discussion

In this study, we employed MS-coupled metabolomics to compare between the metabolomes of tetraploid wheat lines, classified in 4 groups that follow the course of wheat domestication (Fig. 1C). The primary domestication is the evolution of WEW to DEW, whereas the secondary domestication is the evolution of DEW to durum wheat^1^, which is also divided into two groups—LD (i.e., traditional) and MD (i.e., industrial). Three-dimensional (3D) principal component analyses (PCAs), run without a wheat category-bias, also demonstrated a clear separation among the WEW, DEW, and durum accessions in both the embryo and the endosperm tissues as describe here and elsewhere y Ben Abu and Itsko^25^ (Fig. 1). Thus, the heatmaps and the PCAs were consistent with our hypothesis that metabolite composition and expression underwent substantial changes during wheat domestication. Our comparative analysis revealed that many metabolites in wheat kernels had undergone significant changes in each step of domestication. Here, we focused on the specific metabolites of the kernel embryo which show the same tendency of changes during both primary and secondary event of domestication. While many metabolites underwent significant changes, fewer metabolites steadily increased or decreased in association with both domestications events. The output of our comparative metabolomics revealed a multitude of metabolites that were not discussed previously in former metabolomic studies of wheat domestication^21,22^. Notably, we detected a substantial increase in the expression of secondary metabolites that participate in plant-defense mechanisms and response to biotic stress. These compounds include phytoalexins, jasmonic acids, benzoxazinoids, glucosinolates, coumaric acids, and other alkaloids that serve as bactericides, fungicides, and insecticides^30–33^. In a previous report, we characterized the stress response of WEW by producing benzoxazinoids^15,25^. We also detected herbicidal allelochemicals that inhibit the growth of surrounding plants^25,34^. Disease-resistance is one of the most dominant adaptive traits in crop productivity^35^, however, the acquirement of disease-resistance qualities during wheat domestication has not been fully investigated^36^. Few reports have compared thereof the resistance to specific pathogens^37,38^. Interestingly, some of these defense-related metabolites were downregulated when MD lines are compared with LD lines. It is possible that the use of industrial pesticides has led to a loss in the expression of these endogenous pesticides, but not to the extent they are normalized down to the levels found in wild plants. Thus, it could be valuable to improve crop resistance, while minimizing dependence on harmful industrial pesticides^39^. Genetic engineering of crops that promotes the release of phytochemicals that confer resistance to biotic stress may be the technological solution. Genetic regulation of disease resistance in plants is a field of extensive research, however, scientists still need to functionally link between the genes and the bioactive metabolites, so as to genetically improve wheat strains. Several studies have identified certain genes in wheat that promote the expression of defense-mediating metabolites, including metabolites that we associated with wheat domestication^40–42^. Both biotic stress (e.g., pathogens) and abiotic stress (e.g., lack of nutrients, extreme temperatures, and drought) may lead to oxidative stress in plants, against which antioxidants relay protection^43^. Our comparative metabolomics revealed that many antioxidants accumulate in the wheat kernels as an adaptive trait that the domesticated crop most likely acquired. These compounds include cinnamic acid derivatives, apigenin derivatives, flavonols, glutathiones, anthocyanins, and other phenols^43^. Genes that induce expression of antioxidants in wheat may also confer disease resistance^44,45^. Apart from benefits for wheat fitness, high nutritional values and health benefits are attributes of high antioxidant concentrations in plants as a food source^46–48^. Agricultural yield depends on fast and sustained growth of crops under a broad range of environmental conditions as well as on the development of many-fold large grains^49^. The basis for the “green revolution” in the last decades was the introduction of genes that increase crop productivity via phytohormonal regulation^50^. More recent genomic research aims at characterizing the genetic network that contributes to wheat productivity^51–54^. Thus, it is of no surprise that we detected an elevation in plant hormones and their intermediates in domesticated wheat as compared with its primitive and wild progenitors. Additionally, we noticed that amino acid metabolism was significantly altered in the course of wheat domestication, demonstrating an increased proportion of several amino acids. Interestingly only one amino acid (tyrosine) demonstrated decrease during domestication (Fig. 3). Tyrosine is the direct precusor of p-coumaric acid which is a component of defence mechanisms of wheat against biotic and abiotic stresses. Therefore, the relative decrease in level of tyrosine may indicate increased metabolic flux from its pool to that one of p-coumaric acid.

In summary, our metabolomic data can be helpful to identify novel genes that promote the synthesis of defense-related metabolites, antioxidants, and plant hormones; not only in the context of with wheat domestication, but also in the context of genetic engineering of high-yield wheat strains.

## Supplementary Information

Supplementary Information 1.

Supplementary Information 2.

Supplementary Information 3.

## Data Availability

The datasets used and analyzed during the current study are available from the corresponding author on reasonable request.
