# Supplementary material for: Metabolome dynamics during wheat domestication
Source: Sci Rep. 2022 May 20;12:8532. doi: 10.1038/s41598-022-11952-9 (PMC9122938; doi:10.1038/s41598-022-11952-9)
Supplement: Supplementary file 1 — Supplementary Information 1. [file 41598_2022_11952_MOESM1_ESM.pdf]

Figure S1.

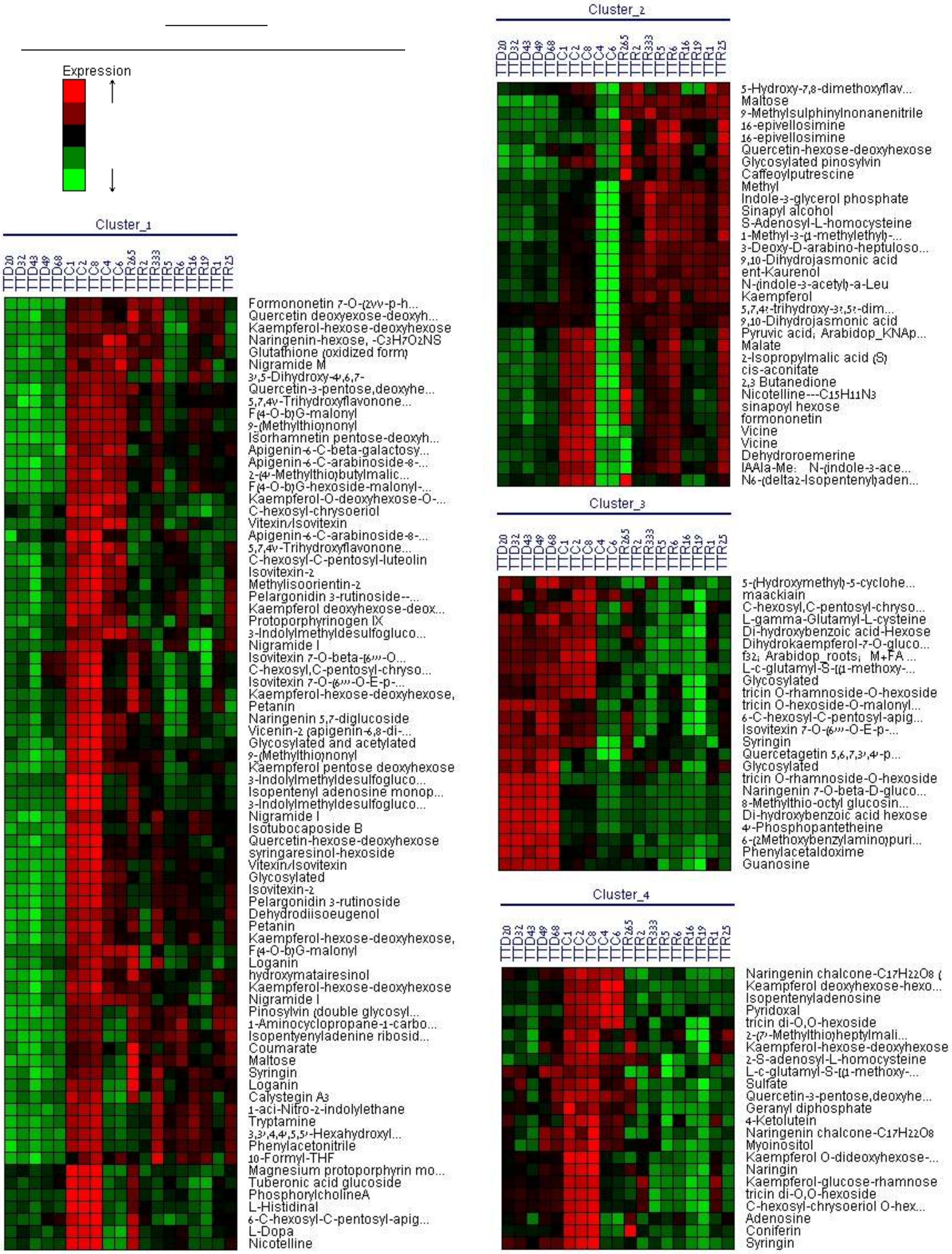

Supplementary Figure 1 (S1). Heat-maps of identified metabolites were generated based on the quantile-normalized average values of embryo metabolites per each wheat line. Four different clusters show co-regulated metabolites. Red color indicates high expression, whereas green color indicates low expression.
