# Supplementary material for: Metabolome dynamics during wheat domestication
Source: Sci Rep. 2022 May 20;12:8532. doi: 10.1038/s41598-022-11952-9 (PMC9122938; doi:10.1038/s41598-022-11952-9)
Supplement: Supplementary file 2 — Supplementary Information 2. [file 41598_2022_11952_MOESM2_ESM.docx]

Supplementary Table 1 (S1): Metabolites that are conserved or steadily changed in association with wheat domestication

| Embryo - Increased | Embryo - Decreased | Embryo - Conserved | Endosperm - Increased | Endosperm - Decreased | Endosperm - Conserved |
| --- | --- | --- | --- | --- | --- |
| 12-Oxo-phytodienoic acid | 1-Methyl-3-(1-methylethyl)-1,2-cyclopentanedicarboxylic acid | 5,10-Methylenetetrahydrofolic acid | Tyramine | Adenosine,5-S-methyl | Solavetivone (two variants) |
| 13(S)-Hydroperoxylinolenic acid | 5-(Hydroxymethyl)-5-cyclohexene-1,2,3,4-tetrol | C-hexosyl-C-pentosyl-luteolin |  | "3,3',4,4',5,5'-Hexahydroxylignan-9,9'-olide | Phenylalanine |
| 16-epivellosimine | 5-Hydroxyconiferyl alcohol | Ferulic acid-fragment |  |  | 1-Piperoylpiperidine; (2E,4E)-form |
| 2-(5'-methylthio)pentylmalic acid | 5-O-Caffeoylshikimic acid | Indole-3-acetaldoxime |  |  | Indole-3-acetic acid |
| 3-hydroxy-4(Z), 6(Z), 8(Z), 10(Z)-tetraenoic acid | 6-(2Methoxybenzylamino)purine-9-beta-D-ribofuranoside | Lucenin |  |  | L-Tyrosine  (two variants) |
| 3-Indolylmethyldesulfoglucosinolate | 6-C-hexosyl-C-pentosyl-apigenin O-hexoside | Nigramide |  |  | Tryptamine |
| 4,8,13-Duratriene-1,3-diol | Coniferyl aldehyde (two variants) | Rutin |  |  | (-)-Phytuberin |
| C15H12O7-C (303)-hexose | Cyanidin 3-O-[2''-O-(xylosyl)-6''-O-(p-coumaroyl) glucoside] 5-O-malonylglucoside |  |  |  | Cycloserine |
| Capsaicin  (two variants) | Di-hydroxybenzoic acid hexose |  |  |  | L-Kynurenine |
| cis-p-Coumaroylagmatine (two variants) | Glycoalkaloid (two variants) |  |  |  | Coumarin |
| Cytidine (two variants) | A glycosylated metabolite? |  |  |  | p-Hydroxybenzaldehyde |
| DIMBOA glucoside (two variants) | Isovitexin 7-O-(6'''-O-E-p-coumaroyl)glucoside |  |  |  |  |
| Ferulic acid | L-Methionine (second variant) |  |  |  |  |
| Feruloylagmatine | L-Tyrosine |  |  |  |  |
| Feruloylserotonin | Pipercyclamide |  |  |  |  |
| Gibberellin | Piperstachine |  |  |  |  |
| Gibberellin A19 | Rishitinol |  |  |  |  |
| Glutamic acid (two variants) | Scopolin |  |  |  |  |
| Glycoalkaloid (two variants) | Sinapaldehyde |  |  |  |  |
| L-Arginine | Sophorol |  |  |  |  |
| L-Dopa |  |  |  |  |  |
| L-gamma-Glutamyl-L-cysteine |  |  |  |  |  |
| L-Kynurenine |  |  |  |  |  |
| L-Methionine (one variant) |  |  |  |  |  |
| L-Valine |  |  |  |  |  |
| Malonylscopolin |  |  |  |  |  |
| N-(indole-3-acetyl)-a-Leu |  |  |  |  |  |
| N-Caffeoylputrescine |  |  |  |  |  |
| Octadecan-1-ol |  |  |  |  |  |
| p-Hydroxybenzaldehyde |  |  |  |  |  |
| Sinapic acid |  |  |  |  |  |
| Sinapyl alcohol (two variants) |  |  |  |  |  |
| Stearic acid |  |  |  |  |  |
| trans-Zeatin riboside monophosphate (two variants) |  |  |  |  |  |
| Undecanoic acid |  |  |  |  |  |
