# Supplementary material for: Metabolome dynamics during wheat domestication
Source: Sci Rep. 2022 May 20;12:8532. doi: 10.1038/s41598-022-11952-9 (PMC9122938; doi:10.1038/s41598-022-11952-9)
Supplement: Supplementary file 3 — Supplementary Information 3. [file 41598_2022_11952_MOESM3_ESM.docx]

Supplementary Table 2 (S2): The most significantly changed metabolites

1. Increased Metabolites (fold-change)

| MD / WE  (>4.0) | LD / WE  (>4.0) | PE / WE  (>5.0) | MD / LD  (>2.0) | MD / PE  (>4.0) | LD / PE  (>5.0) |
| --- | --- | --- | --- | --- | --- |
| cis-Abienol  (9.6, 9.3) | 16-epivellosimine (34.9, 21.5) | DIBOA-hex-hex (30.7) | Quercetin-3-pentose,deoxyhexoside-7-deoxyhexoside (3.0) | L-Glutathione (9.4, 8.0, 6.5) | 16-epivellosimine (21.5, 21.1) |
| DIBOA-hex-hex  (8.2) | cis-p-Coumaroylagmatine (32.5, 27.7) | cis-Abienol  (9.8, 7.3) | Kaempferol-hexose-deoxyhexose, -hexose (2.8) | Syringoylcholine 4-O-hexoside (9.2, 8.7) | cis-p-Coumaroylagmatine (18.7, 8.9) |
| 1,18-Octadecanediol  (8.2, 7.1) | Capsaicin (16.8, 15.4) | 2,3,4,5-Tetrahydrodipicolinate (9.7) | Petanin (2.3) | 6-Dihydrocastasterone (8.3) | Capsaicin (12.6, 9.2) |
| 16-epivellosimine  (7.5, 6.9) | N-Caffeoylputrescine (12.3, 11.2) | Zeatin-9-glucoside-O-glucoside (9.5) | L-gamma-Glutamyl-L-cysteine (2.2) | 16-epivellosimine (7.4, 4.2) | 25-Hydroxyvitamin D3 (10.6) |
| cis-p-Coumaroylagmatine  (6.6, 4.9) | cis-Abienol  (7.8, 6.0) | 6-Methoxybenzoxazolin-2(3H)-one (9.3, 6.7) | L-Glutathione (2.1) | Hexadecane-1,16-dioate (7.1) | L-Glutathione (9.2, 8.8, 5.1) |
| 3-beta-Hydroxysolanascone  (5.6) | 1,18-Octadecanediol (7.0, 4.4) | Spermidine-hydroxyferuloyl-disinapyl (9.0) | DIBOA-hex (2.1) | 16-Hydroxy-hexadecanoic acid (6.2) | Jasmonoyl valine (9.0) |
| Vicenin-2 (apigenin-6,8-di-C-glucoside)  (5.4) | Jasmonoyl valine (5.3) | Vicenin-2 (apigenin-6,8-di-C-glucoside) (8.7) | Naringenin (2.1) | Clusin (5.9, 4.8) | Syringoylcholine 4-O-hexoside (8.7) |
| Hexadecane-1,16-dioate (5.1) | Octadecan-1-ol (5.1) | DIMBOA glucoside (7.8) |  | 3,3',4,4',5,5',9-Heptahydroxy-9,9'- (5.8) | N-Caffeoylputrescine (8.5, 6.6) |
| Octadecan-1-ol (4.9) | Stearic acid (5.1, 4.2) | 1,18-Octadecanediol (7.2, 6.5) |  | 3,3',4,4',5,5'-Hexahydroxylignan-9,9'-olide (5.6, 5.3) | Apo-10'-zeaxanthinal (8.5, 5.0) |
| Quercetin-3-pentose,deoxyhexoside-7-deoxyhexoside (4.8) | Feruloylagmatine (5.1, 4.0) | 1,6-Anhydroglucose (6.8) |  | Bis-coumaroylspermidine O-hexoside (5.6) | Syringoylcholine 4-O-hexoside (8.4) |
| L-Glutathione (4.6) | oxindole-3-acetic acid (5.1) | 3',5-Dihydroxy-4',6,7-trimethoxyflavone (6.7) |  | 3-beta-Hydroxysolanascone (5.3) | 3,3',4,4',5,5',9-Heptahydroxy-9,9'- (7.4) |
| 6-Dihydrocastasterone (4.6) | 3,3',4,4',5,5',9-Heptahydroxy-9,9'-Lignan (4.9) | Coumarin (6.3) |  | 12-Oxo-phytodienoic acid (5.1, 4.0) | Cyanidin 3-O-[2''-O-(xylosyl) (6.9) |
| Stearic acid (4.4) | Phosphorylcholine (4.9) | Apigenin-6-C-arabinoside-8-C-hexoside (6.1) |  | Decanoic acid (5.1) | 6-Dihydrocastasterone (6.5) |
| 9,10-Dihydrojasmonic acid (4.3) | ent-Kaurenol (4.7) | Quercetin-3-pentose,deoxyhexoside-7-deoxyhexoside (6.1) |  | Palmitoleic acid (4.8) | 12-Oxo-phytodienoic acid (6.0, 5.7) |
| Glycoalkaloid (4.3) | DIBOA-hex-hex (4.6) | HBOA-hex-hex (6.0) |  | Cyanidin 3-O-[2''-O-(xylosyl) (4.8) | delta-Tocotrienol (5.7) |
| Capsaicin (4.2, 3.0) | Erythronic acid (4.6) | Phosphatidylethanolamine (5.9) |  | Medioresinol 4'-O-beta-D-glucopyranoside (4.6) | Castasterone (5.6) |
| Octadecene-1,9,18-triol (4.1) | Glutamic acid (4.6) | L-delta1-Pyrroline-5-carboxylate (5.9) |  | L-gamma-Glutamyl-L-cysteine (4.5) | 3,3',4,4',5,5'-Hexahydroxylignan-9,9'-olide (5.5, 5.0) |
| Nigramide I (4.1) | Sinapyl alcohol (4.5) | L-Methionine (5.8) |  | SC(4-O-b)G 4'-O-hexoside (4.4) | oxindole-3-acetic acid (5.1) |
| 16-Hydroxy-hexadecanoic acid (4.1) | Nonanoic acid, 9-(methylsulfinyl) (4.2) | Nigramide I (5.3) |  | 9,10-Dihydrojasmonic acid (4.2) | 5-Hydroxyferuloyl-CoA (5.1) |
| Palmitoleic acid (4.0) | Vicenin-2 (apigenin-6,8-di-C-glucoside) (4.2) | Ribonic acid (5.3) |  | Jasmonoyl valine (4.2) | Hexadecane-1,16-dioate (5.0) |
|  | Glycoalkaloid (4.1) | GDP-L-fucose (5.3) |  | 1,8-Cineole (4.1) |  |
|  |  | Phosphorylcholine (5.1) |  | Protoporphyrinogen IX (4.0) |  |
|  |  | L-c-glutamyl-S-[(1-methoxy-1H-indol-3-yl)methyl]-L-cysteinyl-glycine (5.0) |  |  |  |

1. Decreased Metabolites (fold-change)

| WE / MD  (>3.0) | WE / LD  (>2.0) | WE / PE  (>3.0) | LD / MD  (>3.0) | PE / MD  (>4.0) | PE / LD  (>4.0) |
| --- | --- | --- | --- | --- | --- |
| Di-hydroxybenzoic acid hexose (25.1) | Di-hydroxybenzoic acid hexose (26.1) | Di-hydroxybenzoic acid hexose (17.9) | Octadecene-1,9,18-triol (9.2, 9.0, 4.1) | Spermidine-hydroxyferuloyl-disinapyl (15.5) | Spermidine-hydroxyferuloyl-disinapyl (13.3) |
| Pipercyclamide (17.0) | Cyanidin 3-O-[2''-O-(xylosyl)-6''-O-(p-coumaroyl) glucoside] 5-O-malonylglucoside (13.2) | Glycoalkaloid (12.7, 3.9) | N-Caffeoylputrescine (6.8, 6.6) | Zeatin-9-glucoside-O-glucoside (10.9) | 2,3,4,5-Tetrahydrodipicolinate (10.3) |
| Cyanidin 3-O-[2''-O-(xylosyl)-6''-O-(p-coumaroyl) glucoside] 5-O-malonylglucoside (15.5) | Glycoalkaloid (8.4) | Clusin (10.1) | cis-p-Coumaroylagmatine (6.7, 4.2) | 2,3,4,5-Tetrahydrodipicolinate (8.0) | 6-Methoxybenzoxazolin-2(3H)-one (9.4) |
| Glycoalkaloid (11.8, 4.3) | Clusin (7.3) | Apo-10'-zeaxanthinal (9.3) | ent-Kaurenol (6.6) | 6-Methoxybenzoxazolin-2(3H)-one (7.6) | Zeatin-9-glucoside-O-glucoside (8.3) |
| L-Tyrosine (11.5) | L-Tyrosine (6.0) | 13-apo-b-carotenone (5.9) | Stearic acid (6.2) | Cyanidin 3-O-[2''-O-(xylosyl)-6''-O-(p-coumaroyl) glucoside] 5-O-malonylglucoside (7.6) | DIMBOA glucoside (7.9) |
| Octadecene-1,9,18-triol (9.4) | Pipgulzarine (5.9) | 25-Hydroxyvitamin D3 (5.7) | Capsaicin (5.6, 3.6) | DIMBOA glucoside (6.7) | DIBOA-hex-hex (6.6) |
| Clusin (6.8) | 13-apo-b-carotenone (5.5) | Tricin O-malonylhexoside (5.7) | 16-epivellosimine (5.0) | Kaempferol O-deoxyhexose-hexose-O-deoxyhexose (6.6) | Kaempferol O-deoxyhexose-hexose-O-deoxyhexose (6.5) |
| 13-apo-b-carotenone (6.3) | Pipercyclamide (4.4) | Pipgulzarine (5.7) | Apo-10'-zeaxanthinal (4.6) | Pipercyclamide (6.4) | Cyanidin 3-O-[2''-O-(xylosyl)-6''-O-(p-coumaroyl) glucoside] 5-O-malonylglucoside (6.4) |
| Bis-feruloylspermidine (5.7) | 3,3',4,4',5,5'-Hexahydroxylignan-9,9'-olide (3.9) | L-Tyrosine (4.6) | 18-Hydroxy-9-octadecenoic acid (4.6) | Glycoalkaloid (6.2) | GDP-L-fucose (6.4) |
| Pipgulzarine (5.5) | Tricin di-O,O-hexoside (2.9) | 5-Hydroxyferuloyl-CoA (4.5) | Oleic acid (4.4, 3.4) | L-c-glutamyl-S-[(1-methoxy-1H-indol-3-yl)methyl]-L-cysteinyl-glycine (6.1) | Kaempferol-hexose-deoxyhexose, -hexose (5.7) |
| Apo-10'-zeaxanthinal (5.0) | 6-(2Methoxybenzylamino)purine-9-beta-D-ribofuranoside (2.9) | Bis-feruloylspermidine (3.9) | delta-Tocotrienol (3.9, 3.1) | Alpha tomatine (5.8) | L-c-glutamyl-S-[(1-methoxy-1H-indol-3-yl)methyl]-L-cysteinyl-glycine (4.8) |
| Coumaroyl-N (4.8) | Skimmin (2.7) | 3,3',4,4',5,5'-Hexahydroxylignan-9,9'-olide (3.5) | Pipercyclamide (3.8) | Phosphorylcholine (5.1) | Ribonic acid (4.8) |
| 3,3',4,4',5,5'-Hexahydroxylignan-9,9'-olide (4.1) | Bis-feruloylspermidine (2.5) | Coumaroyl-N (3.2) | Indole-3-acetyl-L-aspartic acid (3.7) | GDP-L-fucose (4.9) | Tricin di-O,O-hexoside (4.6) |
| 18-Hydroxy-9-octadecenoic acid (3.6) | Piperstachine (2.4) | delta-Tocotrienol (3.2) | Methyl jasmonate (3.3) | Dehydrosolanascone (4.7) | Petanin (4.6) |
| Tricin O-hexoside-O-malonylhexoside (3.4) | 5-Methylthiopentanaldoxime (2.3) | Sinapyl alcohol (3.1) | Apigenin-6-C-arabinoside-8-C-hexoside (3.2) | Ribonic acid (4.6) | D-Galacturonic acid (4.6) |
| Coumaroyl- (3.4) | Sophorol (2.2) | Syringoylcholine 4-O-hexoside (3.0) |  | L-Methionine (4.3) | Skimmin (4.4) |
| 6-(2Methoxybenzylamino)purine-9-beta-D-ribofuranoside (3.3) | Isovitexin 7-O-(6'''-O-E-p-coumaroyl)glucoside (2.1) |  |  | Retrofractamide (4.2) |  |
| Isovitexin 7-O-(6'''-O-E-p-coumaroyl)glucoside (3.2) | Tricin O-hexoside-O-malonylhexoside (2.1) |  |  | Chlorophyll b (4.2) |  |
| Oleic acid (3.1) | Coumaroyl- (2.1) |  |  | Octadecene-1,9,18-triol (4.2, 4.0) |  |
| Apigenin-6-C-arabinoside-8-C-hexoside (3.1) | 3beta-Hydroxysolanascone beta-sophoroside (2.0) |  |  | D-Galacturonic acid (4.1) |  |
| Dehydrosolanascone (3.1) |  |  |  |  |  |
| Cyanidin (3.0) |  |  |  |  |  |
